# Selective Enhancing Blood Flow in Solid Tumor Tissue Is the Key for Achieving Satisfactory Delivery and Therapeutic Outcome of Nanodrugs via the EPR Effect

**DOI:** 10.3390/jpm12111802

**Published:** 2022-11-01

**Authors:** Jun Wu

**Affiliations:** Center for Comparative Medicine, Beckman Research Institute of the City of Hope, Duarte, CA 91010, USA; jwu@coh.org

**Keywords:** the EPR effect, tumor blood flow, nanodrugs, solid tumor, cancer targeting drug delivery

## Abstract

The enhanced permeability and retention effect (EPR effect) is a crucial phenomenon for understanding the pathophysiological characteristics of blood vasculature and microenvironments in solid tumors. It is also an essential concept for designing anticancer drugs that can be selectively delivered into tumor tissue via the unique extravasation and retention mechanism for macromolecular drugs. As tumor vasculature is highly heterogeneous, the intensities of the EPR effect vary according to the types and locations of solid tumors in different species. However, the EPR effect is universally observed in a broad spectrum of solid tumors in human cancer as well as experimental animal tumor models. The matter is how to utilize the EPR effect for drug design and clinical application. Many hypotheses were proposed and tested to enhance the EPR effect in solid tumors in order to increase the efficacy of drug delivery. However, we should focus on increasing the blood flow in tumors so that more drugs can be perfused and accumulated inside tumor tissue and execute anticancer activities. Angiotensin II co-administration and the approach of intratumor arterial infusion should be considered to achieve selective tumor tissue perfusion for nanodrugs.

Numerous anticancer compounds have been discovered or created and tested in animal tumor models and clinic cancer patients. Although chemotherapy has achieved significant improvements in prolonging survival time in some types of cancers, cancer remains one of the major killers of human diseases. In recent years, immune cell therapy, such as CAR-T cell therapy, has achieved extraordinarily successful results in some types of hematological malignancies, but CAR-T cell therapy in solid tumors is still far away from satisfactory. These therapeutic agents and immune cells are all facing a fundamental question or obstacle: how to be delivered selectively and specifically into tumor tissue while sparing normal vital tissues and organs in patients. To answer this question, we must take a deep look into the microenvironment of solid tumor, especially the characteristics of tumor vasculature structure and function.

Since early 1940, investigators have started to report their observations on the abnormal vasculature in tumors. We now know that tumor vasculature is highly heterogeneous and abnormal with irregular new blood vessel formation because of angiogenesis, tortuous, poorly organized because of lack of pericytes coverage and normal basement membrane support. The tumor vasculature lacks the conventional hierarchy and responds differently to the blood pressure stimulators. The endothelial cells in tumor blood vessels are discontinuous with unusual gaps between them, which render the leakage to macromolecular agents’ extravasation. The fast growth of tumor compressing against the surrounding matrix forms interstitial fluid pressure. This pressure and abnormal vasculature hamper the blood flow perfusion in tumor tissue. Most times the blood vessels do not have sufficient blood flow, but in intermittent or even reverse actions. The lack of lymphatic vessels in the tumor tissue hinders the recovery and drainage of the macromolecular agents coming out of the tumor tissue. The center of the tumor tissue, thus, becomes hypoxia, acidosis and necrotic due to lack of oxygen and nutrient supply. The hypoxic and acidotic condition in tumor tissue further create a hostile microenvironment which impairs the tumoricidal activities of anticancer drugs and immune cells. Aside from these architectural defects, a lot of inflammatory molecules and cytokines, such as VEGF, HIF-1alpha, nitric oxide, bradykinin and proteases, are involved and orchestrate the campaign of nutrients and oxygen supply for aggressive tumor growth and metastasis [1,2,3].

Maeda and his graduate student Matsumura found that many proteins show the unique behavior of progressive accumulation in tumor tissues during 19 to 72 h. In contrast, small molecules did not achieve such tumoritropic accumulation. They coined the term of the enhanced permeability and retention effect (the EPR effect) to describe this unique functional phenomenon, and advocated a new concept for macromolecular therapeutics in cancer chemotherapy in 1986 [4].

Since the concept was introduced to the field of cancer chemotherapy, it became one of the most cited principles for nanomedicine drug development [5]. However, the clinical therapeutic benefits are insufficient, the inadequate of the EPR effect was questioned [6,7].

Huang and his colleagues did a comprehensive and thorough review on the EPR effect and its related tumor vasculature and microenvironment [8]. They uphold the concept of the EPR effects with extensive evidence from literature. They summarized the factors involving the EPR effect in various tumor models and clinical applications. Several attempts at enhancing the EPR effect for improving the delivery efficacies of nanodrug delivery systems were also discussed.

The key to improving the perfusion and extravasation of anticancer nanodrugs into tumor tissue is to improve the blood flow into tumor tissues. Vascular normalization is one of the hypotheses trying to address the issue of poor blood perfusion into tumor tissues [9]. It is believed that the drugs and oxygen cannot be pumped into tumor tissue if the tumor vasculature is abnormal, thus, temporarily normalizing the tumor vasculature for facilitating drug delivery to become a rational temptation. Normalization of tumor blood vessels with a low dose of antiangiogenic antibodies did improve the uptake of smaller nanoparticles under 12 nm, but hindered the uptake of nanoparticles larger than 125 nm [10]. In order to achieve the benefits of functional vascular normalization, the selection of doses of antiangiogenic drugs and the timing of administration is crucial [11]. Since the administration of antiangiogenic antibodies will significantly reduce the tumor blood flow and decrease vessel pore size, the uptake of trastuzumab was hampered even with the carefully designed dose and regimen of bevacizumab in mouse xenograft models of human ovarian and esophageal cancer [12,13]. In a randomized clinical trial, bevacizumab was given with a standard oxaliplatin regimen in patients with metastatic colorectal cancer. The primary end-point objective response rate did not significantly differ between the sequential and the concomitant in combination with standard oxaliplatin regimen [14]. The normalization window is transient and how to find out the optimal dose and timing for individual patients to achieve personalized tumor vascular normalization still remains unanswered [15].

Suzuki and colleagues at Tohoku University, Japan, found out that by administration of angiotensin II, the tumor blood flow can be selectively enhanced up to 5.7 folds while without increasing blood flow in normal tissue. The therapeutic outcome of mitomycin C chemotherapy on both main tumor and lymph node metastatic foci was significantly improved. In 1981, they advocated a new approach to cancer therapy of combining angiotensin II to enhance chemotherapeutic drugs delivered into tumor tissue via selectively enhancing tumor blood flow [16]. Interestingly, angiotensin II enhances tumor blood flow regardless of the increase in interstitial fluid pressure [17]. They even discovered the circadian fluctuation in tumor blood flow and pointed out that the blood flow rate is twice higher in the nighttime zone compared with the daytime zone. Angiotensin II administration can further induce higher tumor blood flow during the nighttime zone. Their findings suggested that nanomedicine for cancer therapy should be administered in the nighttime zone with the enhancement of angiotensin II administration [18]. The selective enhancement of tumor blood flow by angiotensin II is obvious and straightforward compared to the sophisticated method of balancing pro- and antiangiogenic factors in order to achieve transient vascular normalization for drug delivery. However, unfortunately, no nanodrug was reported to be administered into the tumor combined with angiotensin II administration. The advantage of angiotensin II enhancing tumor blood flow has been largely and astonishingly ignored by the nanomedicine research community.

Tumor vasculature is highly heterogeneous, and the tumor blood flow is also highly heterogeneous, thus, the intensities of the EPR effect certainly vary depending on the types and locations in human and animal cancer patients. Nonetheless, the concept of the EPR effect has a profound impact for our understanding of the molecules extravasating across the tumor vasculature and solid tumor tissue. It may be ideal to find out clinical markers or assays to evaluate the scores of the EPR effect in individual cancer patients to guide personal chemotherapy.

Tumor is fed by arteries and tumor vasculature is branching out mainly from the terminal arteriole [19]. Excellent hepatocellular carcinoma chemotherapy via tumor-feeding artery infusion was successfully established in early 1980s [20,21]. Together with the improvement of imaging technology, intervention radiology has been prevailing in cancer therapy in clinics [22]. However, the drugs used in interventional oncology are not nanodrugs, but conventional small molecule chemo drugs. In 2016, Jeon et al. reported that doxorubicin-loaded porous magnetic nano-clusters with iodinated oil administered by intra-arterial infusion method achieved excellent retention and anticancer effect in rabbit liver tumor tissue [23]. It is again to remind us that it is imperative to combine angiotensin II (enhance tumor blood flow) with nanodrugs (better functional delivery) via the approach of interventional therapy through the tumor feeding artery (selective and localized delivery) in experimental animal tumor models and clinical practice.
